# Distributed acoustic cues for caller identity in macaque vocalization

**DOI:** 10.1098/rsos.150432

**Published:** 2015-12-23

**Authors:** Makoto Fukushima, Alex M. Doyle, Matthew P. Mullarkey, Mortimer Mishkin, Bruno B. Averbeck

**Affiliations:** Laboratory of Neuropsychology, National Institute of Mental Health, National Institutes of Health, Bethesda, MD 20892, USA

**Keywords:** animal vocalization, macaque, voice recognition, caller identity

## Abstract

Individual primates can be identified by the sound of their voice. Macaques have demonstrated an ability to discern conspecific identity from a harmonically structured ‘coo’ call. Voice recognition presumably requires the integrated perception of multiple acoustic features. However, it is unclear how this is achieved, given considerable variability across utterances. Specifically, the extent to which information about caller identity is distributed across multiple features remains elusive. We examined these issues by recording and analysing a large sample of calls from eight macaques. Single acoustic features, including fundamental frequency, duration and Weiner entropy, were informative but unreliable for the statistical classification of caller identity. A combination of multiple features, however, allowed for highly accurate caller identification. A regularized classifier that learned to identify callers from the modulation power spectrum of calls found that specific regions of spectral–temporal modulation were informative for caller identification. These ranges are related to acoustic features such as the call’s fundamental frequency and FM sweep direction. We further found that the low-frequency spectrotemporal modulation component contained an indexical cue of the caller body size. Thus, cues for caller identity are distributed across identifiable spectrotemporal components corresponding to laryngeal and supralaryngeal components of vocalizations, and the integration of those cues can enable highly reliable caller identification. Our results demonstrate a clear acoustic basis by which individual macaque vocalizations can be recognized.

## 1. Background

We recognize others by their voices, and brain damage can lead to deficits in voice recognition ability (phonagnosia) [1,2] in humans. Cases of developmental phonagnosia [3] have demonstrated a modality-specific deficit in voice identity recognition despite normal understanding of speech and no identified pathological abnormalities in the brain [4]. These cases are consistent with the idea that voice recognition is mediated by a mechanism separated from speech processing [5]. Non-human primates, lacking speech, have also demonstrated an ability to identify individuals from their voices [6–10]. For example, macaques have demonstrated an ability to discern conspecific identity from ‘coo’ calls [11–14], which are one of the most common calls produced by this species.

Voice recognition presumably requires integrating low-level acoustic features to obtain a holistic representation of voice, as is the case with face recognition in vision [5,15]. Previous studies suggest that acoustic cues are available in multiple aspects of coo calls for an animal to achieve caller identification [12,13]. However, the amount of caller identity information available in various single and combined acoustic cues of macaque coo calls has not been determined by factoring in the substantial variability across utterances. Thus, we tested the hypothesis that highly reliable acoustic information is distributed in spectral and temporal scales of the calls for caller identification. To estimate variability and consistency of caller information across utterances in a high dimensional parameter space, this hypothesis needed to be tested with a large sample of calls from multiple animals. In this study, therefore, we set out to examine caller identity information in hundreds of calls from each of eight macaques using advanced statistical techniques.

## 2. Material and methods

### 2.1 Subjects

We used eight adult rhesus macaques: four females (monkey Tw, Th, Io, Sn) and four males (monkey Be, Al, Qu, Mu), weighing between 4.58 and 8.45 kg (table 1).

**Table 1.** The number of coo call samples and body weights for each animal. Each animal’s gender is indicated in parentheses. Body weight was estimated from the average of two to three measurements.

monkey ID	weight (kg)	call sample size
Al (male)	8.45	999
Be (male)	8.05	478
Qu (male)	4.9	975
Mu (male)	5.7	1017
Io (female)	4.58	1002
Sn (female)	8.2	1001
Th (female)	4.75	1345
Tw (female)	5.8	468
total		7285

### 2.2 Call recording

Animals were selected for having high spontaneous vocalization rates for coo calls in either their home cage or testing room [16]. For call recording, we trained vocal animals to emit coo calls frequently in a sound-attenuating booth, by providing a juice reward after each coo call was issued. Training started by rewarding calls with dry treats or fruit in their home cage and later in a chair outside of the cage. In subsequent stages of training, the animals were taken to the testing room, and gradually the dry treats and fruit were replaced with water or apple juice reward. At this point, the animal’s daily water allotment was gradually controlled. After the animals were taking liquid reward from a water bottle for the calls they produced, they were transitioned to taking liquid from a steel mouthpiece inside a sound-attenuating booth. During call recording, we provided a small amount of juice (approx. 5 ml) after each coo call produced. All coo calls were rewarded equally. We recorded the call with a directional microphone (Sennheiser ME66; frequency response 0.04–20 kHz, ±2.5 dB) at a sampling rate of 50 kHz.

### 2.3 Semi-automatic call segmentation

We used a custom-written program in Matlab (MathWorks) for segmenting calls from continuous sound recording, based on a method used in a previous study [17]. We first calculated the amplitude envelope of the sound, by applying a band-pass filter (0.1–8 (kHz)) to sound waveforms, followed by full-wave rectification and a low-pass filter at 200 Hz. The amplitude envelope was then transformed to a logarithmic scale. We determined the mean background noise level with a peak in the sound level distribution estimated by kernel density estimation. The full width at half maximum of this sound level distribution was the estimated standard deviation (s.d.). Finally, periods containing calls were detected by setting a sound threshold at the mean background noise level +4 s.d. We then confirmed that the segmented sounds were coo calls to exclude non-call sounds and calls in other call categories.

### 2.4 Acoustic features

For each of the segmented calls, we calculated the following six acoustic features: fundamental frequency (MF0), duration (Dur), zero crossing rate (ZCR), maximum frequency (maxF), Wiener entropy (WE) and mean frequency (MF). MF0 was calculated by averaging the time course of the fundamental frequency of the segmented call over time. The fundamental frequency was estimated using TANDEM-STRAIGHT ([18], http://www.wakayama-u.ac.jp/~kawahara/STRAIGHTadv/index.e.html), which is designed to detect F0 in human speech. For detecting F0 in macaque coo calls, we set the search range of F0 in STRAIGHT to 400–800 Hz (for monkey Sn) or 200–800 Hz (for the seven other monkeys). Dur was simply calculated by the sample size of the segmented call multiplied by the inverse of the sampling frequency. ZCR was calculated by counting the number of zero crossings from positive to negative and dividing by the Dur. maxF, MF and WE were estimated from the power spectrum of the call. The power spectrum was calculated by the multi-taper method with the time–bandwidth product = 3.5. maxF was calculated as the frequency at which the power spectrum peaked. MF was the arithmetic MF weighted by the power. WE was defined as the logarithm of the ratio between the geometric and arithmetic means of the power spectra [19]. WE quantifies the flatness of the power spectrum, and thus it is also called spectrum flatness [20]. It quantifies how uniformly the power is distributed across frequencies in the power spectrum.

To evaluate the similarity among the distributions of the acoustic features, we calculated pairwise correlation coefficients among the six features and used these to form a dissimilarity matrix for visualizing the similarity among features using multidimensional scaling (MDS). MDS was carried out with classical scaling (‘cmdscale’ function in Matlab).

### 2.5 Modulation power spectrum

The modulation power spectrum (MPS) quantifies spectral and temporal modulations of the sound spectrogram. MPS is defined as the amplitude spectrum of the two-dimensional Fourier transform of the spectrogram [21,22]. Because the units for the dimensions of the spectrogram are the temporal frequency (kHz) and the time (s), the corresponding units for two-dimensional frequencies of the MPS are the spectral modulation (cyc kHz^−1^) and the temporal modulation (Hz). We estimated the MPS for each call following the procedure used in a previous study [23]. The range of temporal modulation was ±200 (Hz), sampled with 99 points. The range of spectral modulation was 0–10 (cyc kHz^−1^), sampled with 50 points.

### 2.6 Classification analysis

To evaluate the caller identity information of calls, we used the following two statistical classifiers. Chance performance was 12.5% (=1/8) in both cases.

#### 2.6.1 Classifier for acoustic features

For the classification of single or combined acoustic features (three features; MF0, Dur, WE), we used a linear classifier estimated from a multivariate normal density with a pooled estimate of covariance (‘classify’ function in Matlab). Each feature was standardized to have zero mean and unit variance. The dimensionality of the predictor variable was either 1 (single feature) or 3 (combined features). To evaluate the caller identity information with uniform sample size across animals, we randomly chose 400 calls from each animal (total 3200 calls) and split them into a training set (three-quarters of the calls) and a validation set (one-quarter of the calls). The training set was used to estimate parameters of the linear classifier. Then, this classifier was used to classify calls from the validation set. The percentage of calls correctly classified in the validation set was used as the measurement of classification performance. We repeated the analysis by choosing 100 different sets of 400 calls randomly and used the mean performance as the estimate for the classification performance.

#### 2.6.2 Classifier for modulation power spectrum

The dimensionality of the predictor variable for MPS was 5445 (=99 (temporal modulation) × 50 (spectral modulation)). This is higher than the dimensionality of the acoustic features or the call sample size for each animal. Thus, we estimated a classifier using the early-stopping algorithm that instantiates a type of regularization [24]. This classifier consists of a multinomial regression model with a softmax-link function for estimating the posterior probability of the caller, given the MPS of a call. Parameters were estimated by maximizing the log-likelihood using cross-validated early stopping, with a procedure similar to that used in our previous study [25]. We first chose 450 calls from each of eight animals randomly. The dataset was then divided into the following three sets: a training set (two-thirds of the calls); a validation set (one-sixth of the calls) and a stopping set (one-sixth of the calls). The training set was used to update the parameters such that the log-likelihood was improved on each iteration through the data. The stopping set was used to decide when to stop the iterations on the training data. The iteration was stopped when the log-likelihood function value calculated with the stopping set became smaller than the value in the previous iteration. At this point, performance no longer improved on the stopping set. Then, this classifier was used to classify calls from the validation set. We repeated this for all six possible divisions of the data. The percentage of calls correctly classified in all validation datasets was then used as the measurement of classification performance. We repeated this analysis by choosing 1000 different sets of 450 calls from the eight animals randomly. The mean classification performance was then used as the estimate of the classification performance.

The parameters of the multinomial classifier are associated with each of the 5445 points of the MPS. The magnitude of these parameter values quantifies the degree to which the classifier used each point in this space to identify the caller. As each parameter value was estimated 1000 times, we determined that the magnitude of the parameter value was significantly different from zero when more than 99.5% of the 1000 estimates were larger than zero.

We also did the same classification analysis using a restricted region of the MPS as the predictor variable. We chose the area in the MPS with the range of spectral modulation of 0–1.2245 (cyc kHz^−1^) and the temporal modulation of ±16.3265 (Hz). The maximum spectral modulation in this area corresponds to harmonics with a peak-to-peak separation of 816.7 (Hz), which is higher than the fundamental frequency produced by any of the eight animals. This ensures that the small MPS region does not include a spectrum modulation component resulting primarily from harmonics of the fundamental frequency.

### 2.7 Multidimensional scaling from the confusion matrix of the classifier

MDS allows for converting dissimilarity among pairs of samples to distances between points of a low-dimensional multidimensional space [26]. We calculated classical MDS from the dissimilarity matrix derived from the confusion matrix of the classifier. It is standard practice to convert the confusion matrix (which is an asymmetrical matrix in general) to a similarity matrix by averaging the upper and lower triangular parts and setting the diagonal values to one [27]. By converting this to the dissimilarity matrix (1 – similarity values), the classical MDS plots each animal in the coordinates of the space. The MDS dimensions were sorted by the eigenvalues from the matrix of the scalar product of the MDS coordinate values. The first MDS dimension corresponds to the highest eigenvalue.

## 3. Results

**Figure 1.**
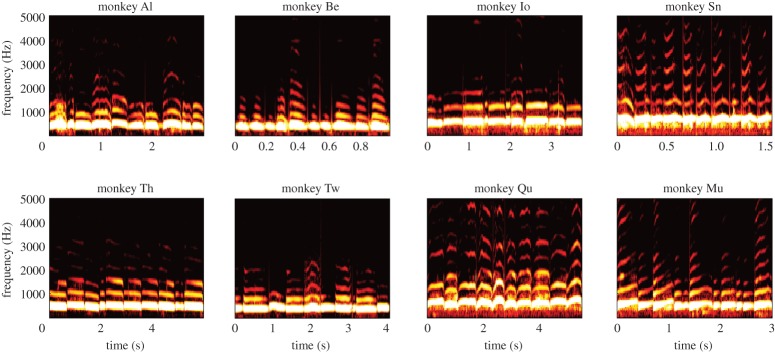
Spectrograms for 10 coo calls from each of eight monkeys (monkeys Al, Be, Io, Sn, Th, Tw, Qu and Mu). Each plot shows 10 calls. Each of 10 calls was recorded separately and selected randomly from all samples. Those calls were then concatenated and shown as one spectrogram.

A total of 7285 coo calls from eight animals were used for the analysis (table 1). We examined example call spectrograms (figure 1) for 10 concatenated coo calls from each of eight animals. Inspecting these examples shows the extent of variability across call samples within each animal, and the consistency across the call examples that differs among animals. In the following, we quantitatively evaluated how much information was available in coo calls for caller identification across variable utterances.

### 3.1 Single acoustic features for evaluating caller identity

**Figure 2.**
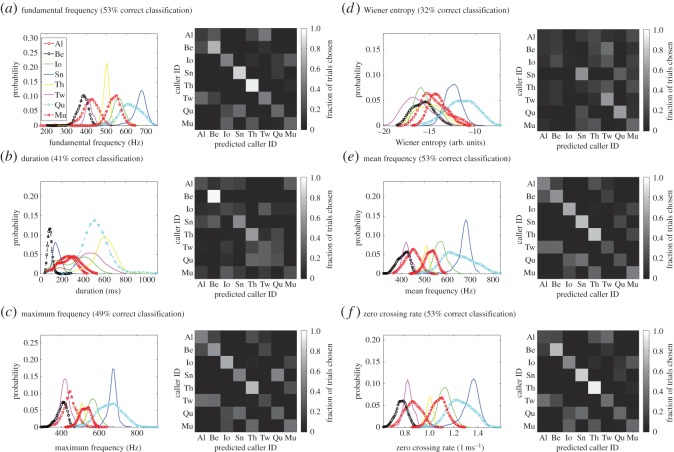
Classification analysis to identity the caller from a single acoustic feature. The results for the following six acoustic features are shown: (*a*) fundamental frequency (MF0), (*b*) duration (Dur), (*c*) maximum frequency (maxF), (*d*) Wiener entropy (WE), (*e*) mean frequency (MF) and (*f*) zero crossing rates (ZCR). For each feature, the left panel shows the probability distributions of an acoustic feature for eight animals. The distributions for males (Al, Be, Qu and Mu) were plotted by open symbols with dotted lines, whereas those for females were plotted with closed symbols with solid lines (Io, Sn, Th and Tw). The right panel is a graphical representation of the confusion matrix from the classification analysis. The average performance for classification is also shown for each feature. The chance level for the classification is 12.5% (=1/8).

We first quantified the acoustic variability of coo calls using acoustic features commonly used for characterizing animal vocalizations. The following six acoustic features were used; mean fundamental frequency (MF0), duration (Dur), maxF, WE, MF and ZCR (see Material and methods). We plotted the probability distribution of each acoustic feature for each of eight animals separately (left panels in figure 2). We then used a linear classifier to evaluate the caller identity information in each acoustic feature. The classifier quantifies how well the distributions are separated among animals. The larger the separations, the easier it is to identify the caller from a given acoustic feature value. The confusion matrix shows the fraction of correct or incorrect predictions made by a classifier for each animal separately (right panels in figure 2*a*). Diagonal elements of the matrix show the fraction of correct predictions. Thus, a high accuracy in the classification is reflected in a clear diagonal line in the matrix plot.

MF0 is an acoustic feature that characterizes the fundamental frequency or the pitch of harmonics. MF0 values from all animals are distributed between 300 and 750 (Hz) (left panel; figure 2*a*). There is a notable difference among animals in terms of the degree of the width of the distribution and the amount of overlap among the distributions. Monkey Th has a narrowly peaked distribution (yellow line; figure 2*a*, left panel), and thus it would be easy to distinguish this animal from other animals, relying on MF0 values. Accordingly, the confusion matrix of the linear classifier shows the best performance for monkey Th (figure 2*a*, right panel). The second best performance was obtained for monkey Sn, which has the highest MF0 (approx. 700 Hz). This has little overlap with distributions from most of the other animals (dark-blue line; figure 2*a*), except for monkey Qu (light-blue line; figure 2*a*). The mean classification performance from MF0 across the eight animals was 53%.

The feature distributions for MF0 were similar to the following three acoustic features, maxF (figure 2*c*), MF (figure 2*e*) and ZCR (figure 2*f*). These three features also provided classification performances (49–53% correct classification) similar to that for MF0. The pattern of errors was also similar among these features, as can be seen from the confusion matrices (right panels in figure 2*a*,*c*,*e* and *f*). On the other hand, the feature distribution and confusion matrix for MF0 were very different from those for Dur (figure 2*b*) and WE (figure 2*d*). Dur was less informative about caller identity on average (41% correct classification, respectively; figure 2*b*) than was MF0. In fact, the correct classification is mostly from one animal (monkey Be, 85% correct classification). Accordingly, monkey Be had a sharply peaked distribution with a mean short duration (black dotted line; figure 2*b*, left panel), well separated from the distributions of all other animals. WE was another feature that was distributed differently from MF0. The mean classification performance was 32% correct.

**Figure 3.**
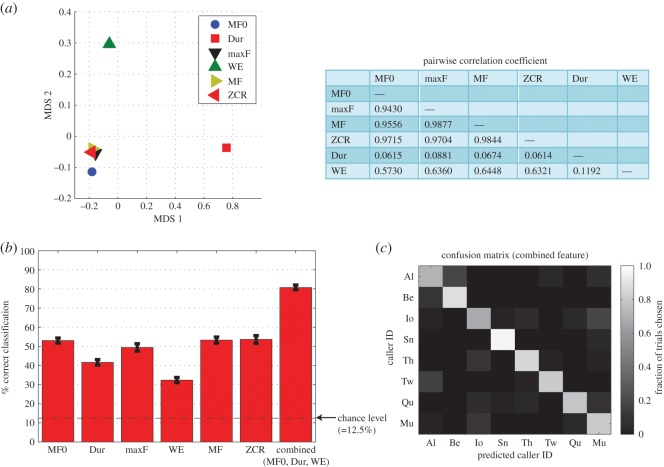
Classification analysis from single and combined acoustic features. (*a*) Multidimensional scaling of six acoustic features. Left: the first two dimensions of MDS are plotted for the six features. The pairwise correlation coefficients (table shown on the right) were used as the dissimilarity metric (i.e. pairwise distance) for MDS. (*b*) Classification performances (% correct) for each of the six single features in figure 2, and for the combined feature (MF0, Dur and WE). Mean±s.d. for 100 repeated analyses with random sampling (see Material and methods). (*c*) Confusion matrix for the classification analysis for the combined feature. The mean classification performance was 80%.

We explicitly evaluated the similarity among acoustic features by calculating pairwise correlation coefficients among six acoustic features from all call data (figure 3*a*, right panel). The pairwise correlation values were very high among MF0, max F, ZCR and MF (0.943–0.9877), whereas they were lower between those features and Dur or WE. We used MDS on the transformed correlation matrix to visualize the similarity among the six features. The MDS plot of the first two dimensions clearly showed clustering of the four acoustic features (MF0, maxF, MF, ZCR), whereas Dur and WE were located distantly from the others (figure 3*a*, left panel).

### 3.2 Combination of acoustic features for caller identification

Results from single acoustic features suggest that (i) statistical classifiers from single acoustic features contain significant caller identity information and (ii) information from some features are redundant but those from others are not. Thus, the caller identity cues are distributed across acoustic features, and combining them could provide more information about identity.

We indeed found that a classifier using combined features outperformed single acoustic features. As we examined above, the four acoustic features (MF0, maxF, MF, ZCR) provided redundant acoustic information for the call (figure 3*a*). We therefore chose one of those four features (MF0) and combined it with the other two features, i.e. the three-dimensional vector (MF0, Dur, WE) was used as the predictor variable for the classification analysis. The mean classification performance from this classifier was 80%, which is higher than the performances from each of the three individual features (32–53%; figure 3*b*,*c*). These results suggest that the caller identification can be improved by combining acoustic features distributed across different spectral (MF0 and WE) and temporal (Dur) aspects of coo calls.

### 3.3 Caller identification from the modulation power spectrum

**Figure 4.**
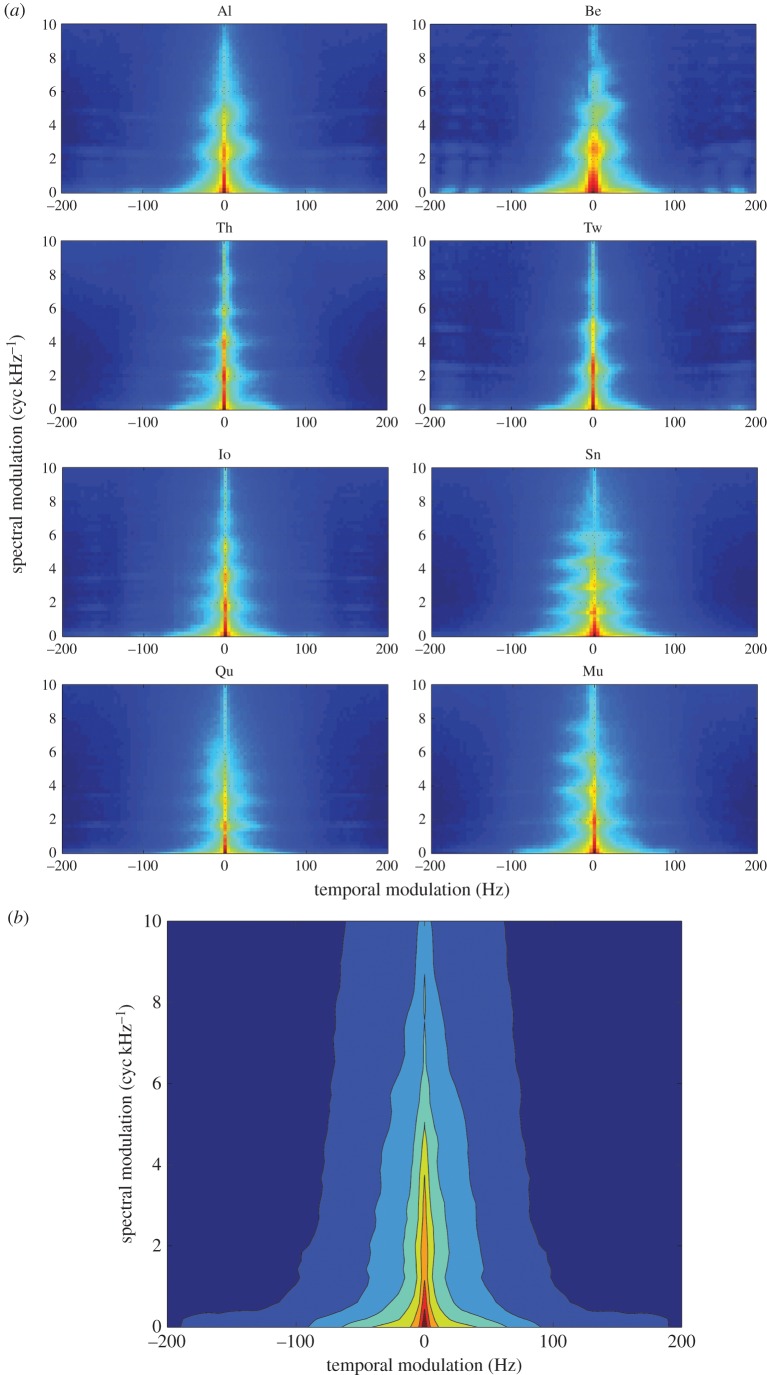
Modulation power spectrum (MPS) of the coo call. (*a*) Averaged spectrum for each of the eight animals across call samples. Monkey ID is shown above each panel. (*b*) Averaged spectrum across the eight animals.

**Figure 5.**
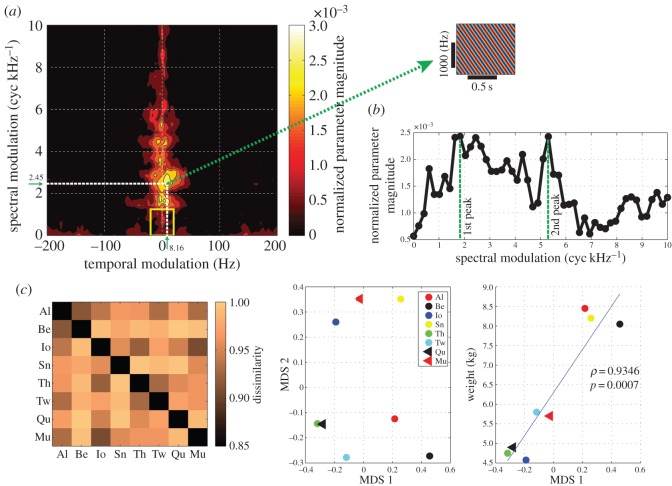
Classification analysis from the call MPS. The mean classification performance was 92%. (*a*) Normalized magnitude of the parameter in the softmax-link function of the multinomial classifier, averaged across the eight animals. They are normalized by the total magnitudes for each animal and then averaged across animals. The yellow square indicates the region with the range of spectral modulation of 0–1.2245 (cyc kHz^−1^) and the temporal modulation of ±16.3265 (Hz). Inset: the plane wave corresponds to the peak spectral and temporal modulation (2.45 (cyc kHz^−1^), 8.16 (Hz), indicated by the white dotted lines in the magnitude profile). (*b*) The normalized magnitude of the classifier coefficient at zero temporal modulation frequency is shown as a function of the spectral modulation frequency. The data values are the same as in those in (*a*). The first peak of this curve is located at 1.84 (cyc kHz^−1^), and the second peak is located at 5.30 (cyc kHz^−1^). (*c*) Classification analysis from the restricted component of MPS, indicated by the yellow square in (*a*). The mean classification performance was 73.6%. Left: the dissimilarity (or distance) matrix derived from the off-diagonal component of the confusion matrix in the classification analysis. Centre: the first two dimensions of MDS derived from the dissimilarity matrix on the left. Right: animal body weights as a function of the first dimension of MDS. The correlation coefficient between those two variables was 0.9346 (*p*=0.0007). The solid line indicates the linear regression.

We have thus far examined the degree to which predefined acoustic features allow for caller identification. In the next analysis, we took a data-driven approach to identifying acoustic features informative for caller identity. We represented each call with the MPS to quantify the spectral and temporal modulations of the call spectrogram [21–23] (Material and methods). The difference in the MPS among animals can be seen from the MPS averaged across call samples for each of eight animals (figure 4*a*). The average MPSs showed distinct shapes for each monkey, with a shared triangular-shaped power distribution, which is also visible in the MPS averaged across animals (figure 4*b*). To determine which part of the MPS is informative for caller identity, we trained a regularized classifier to identify the caller using MPS as the predictor variable (Material and methods). The mean classification performance from this classifier was 92%, which is quite high compared with the results from predefined acoustic features. Now, by examining the magnitude of parameters from the classifier (figure 5*a*, left panel), we were able to determine which spectral and temporal regions of the MPS contained caller identity information. The spatial pattern of the parameter magnitude (figure 5*a*, left panel) was quite different from the averaged MPS (figure 4*b*). In other words, only restricted regions of the MPS were informative for caller identity. Areas of high amplitude (figure 4*b*) but not high information (figure 5*a*) either did not differ on average among callers, or had significant variability within individual callers. In particular, the classifier placed less emphasis on lower spectral modulation with higher temporal modulation, lacking the triangular shape profile notable in the averaged MPS.

The informative parts of the MPS correspond to acoustic features such as the fundamental frequency helpful in discriminating identity. The profile of the classifier coefficient at 0 (Hz) temporal modulation (figure 5*a*, right panel) corresponds to a ‘plane wave’ in the spectrogram, whose spectral modulation is parallel to the frequency direction in the spectrogram (cf. [23]). For example, a harmonic sound can be approximated to such a ‘plane wave’ in the spectrogram. The first peak of the parameter magnitude profile at zero temporal modulation was 1.84 (cyc kHz^−1^) (figure 5*b*). We can interpret this in terms of the ‘plane waves’ in the spectrogram. The value corresponds to the peak-to-peak separation of 544 (Hz) in the plane wave. For a harmonic sound such as a coo call, this peak-to-peak separation is equivalent to the fundamental frequency, because the frequencies of harmonics are multiples of the fundamental frequency. Thus, this means that the fundamental frequency values around 544 (Hz) was helpful in discriminating callers. In fact, this value is located in the midst of distributions for the fundamental frequency (figure 2*a*). Therefore, the classifier identified a region of the MPS that corresponds to the fundamental frequency helpful for identifying the caller.

We also noted that the parameter profile of the classifier (figure 5*a*, left panel) was peaked at an off-centre location of the MPS. The peak location of the entire profile was located at the point with spectral modulation of 2.45 (cyc kHz^−1^) and temporal modulation of 8.16 (Hz) (figure 5*a*; left panel, indicated by the dotted white lines). This off-centre point corresponds to a downward FM sweep (or tilted plane wave) in the spectrogram (figure 5*a*, indicated by the green arrow). Therefore, this suggests that we should be able to differentiate animals from the degree of downward FM sweep in coo calls. In fact, the FM sweep directions in coo calls were different among the animals (figure 1). For example, monkey Be tends to produce a downward FM sweep call, whereas monkey Mu had upward FM sweep calls. Monkey Sn tends to have both up and down FM sweeps over time. These observations confirmed that the amount of downward FM sweep was a helpful feature for identifying the callers.

In sum, by training a classifier to identify callers from a large sample of the call MPS data, we determined spectrotemporal modulation scales that correspond to signature features for individual macaque voices.

### 3.4 Caller identification from the low-frequency component in modulation power spectrum

The area with low spectrotemporal modulation corresponds to coarse spectral amplitude modulations, formed by the upper vocal tract filter rather than by the sound source component (e.g. fundamental frequency). The low spectrotemporal modulation component could correspond to formants [23]. As macaques have demonstrated behavioural sensitivity to formants [28], we examined how much information could be extracted from just the small region of the MPS with low spectrotemporal modulation. To do so, we chose the area in the MPS delineated by the yellow box in the profile (0–1.2245 (cyc kHz^−1^), ±16.3265 (Hz); figure 5*a*). We then trained a classifier to identify the caller using only this area of the MPS (Material and methods). The mean performance of this classifier was 73.6%, suggesting that this small area of low spectral and temporal modulation contained significant information about caller identity.

Could this small MPS region contain any cues for physical characteristics of the caller (i.e. indexical cues)? It has been suggested that formants of calls in macaques contain the indexical cues for the caller, in particular body size [13,29]. If so, then we should be able to find a relationship between an animal’s weight and the similarity among animals judged by the classifier trained on this MPS region. Because the classifier made a significant amount of errors (approx. 26.4%), we can use the pattern of the errors in the confusion matrix as a metric of the similarity among animals. In other words, if a particular animal was often misclassified as another animal, then they are similar to each other for the classifier. Thus, we carried out classical MDS on the dissimilarity measurements among animals, derived from the confusion matrix of the classifier (see Material and methods). This allowed us to compare each animal’s spatial position, reflecting the similarity from the small MPS area, with the absolute (not relative) metrical measurement of body weight (table 1). Interestingly, we found that the first dimension of the MDS was highly correlated with the animal’s body weight (*ρ*=0.9346,*p*=0.0007). This result demonstrates that the MPS contained information about caller identity that correlated with animal’s body size. Thus, the spectrotemporal scales informative for the caller associated with supralaryngeal component (i.e. upper vocal tract filter) contained the indexical cue of the caller.

## 4. Discussion

Our results both from predefined acoustic features and the data-driven approach with the MPS identified multiple distributed acoustic cues for caller identity in macaque coo calls. We found that combining those multiple cues enabled highly reliable caller identification. Furthermore, our data suggest that a specific part of the call MPS contained an indexical cue of the caller body size. Significant information for caller identity had been identified in acoustic features of the coo calls in previous studies [12,14,30]. However, our large sample size and data-driven analysis approach allowed for exploring the parameter space of caller identity cues in its full detail, factoring in the substantial variability across utterances within each individual macaque.

Sound components of vocalizations can be described with the source-filter model for vocal production, consisting of the laryngeal (source) and supralaryngeal (upper vocal tract filter) components [31]. The high spectral modulation features (e.g. MF0, FM sweep direction) could correspond to the laryngeal component whereas the low spectrotemporal modulation features (e.g. formants) would correspond to the supralaryngeal components. Our results showed that both of these features contained caller identity cues. Previous studies have suggested that both laryngeal and supralaryngeal components of vocalizations are critically involved in voice perception. Human listeners have difficulty in recognizing voices when either of these components is manipulated [32–34]. Macaques have demonstrated an ability to discriminate pitch [35–37], a component of the vocal source, and to use it for the caller discrimination [14]. They also perceive changes in the formant frequency (i.e. supralaryngeal component) in both trained [38] and untrained animals [28]. Macaques can associate formants with body size, suggesting that they can extract an indexical cue from acoustic voice information [13]. Here, we found the supralaryngeal component, corresponding to the low spectrotemporal modulation component, was correlated with the animal’s body size. This finding was also consistent with a previous study that found a correlation between the formant frequency dispersion and body size [29].

Although our analysis detected multiple acoustic features statistically informative for caller identification, further behavioural testing would be required to identify which of these acoustic features contribute to perception of caller identity. To evaluate the behavioural relevance of an acoustic feature for caller identification, one could use synthetic sounds in which one changed or eliminated a particular acoustic cue. For example, we could make an acoustic feature (e.g. duration) identical or similar to all calls with other features preserved to see how much the animal’s caller identification performance is altered for constant duration stimuli [14]. To examine the behavioural relevance of features defined by regions of the modulation spectrum, the synthetic sounds could be created by filtering out a particular domain of spectrotemporal modulation frequency [23]. Furthermore, our results here show that combinations of acoustic features provide better caller identification than a single acoustic feature. To examine whether specific combinations of features affect caller identification behaviourally, one could create stimuli that have particular acoustic feature values recorded in natural calls but with a combination that never occurs in natural calls.

Our call recordings were carried out in an experimental setting, and calls emitted in a different context could provide us a different amount of caller identity information. Macaques may alter the acoustic structure of coo calls in different contexts [39,40]. These alterations may even arise from the engagement of different brain regions during vocal production [16,41,42]. Thus, it is possible that the amount of caller identity information could also depend on the context, although some features would remain constant because of constraints from the physical characteristics of callers (e.g. vocal tract length). In a previous study using coo calls recorded from free-ranging macaques [12], statistical classification performance from various acoustic features was 63–79% correct for classifying 16–54 coo calls from each of 17 macaques. This performance level is higher than our result from a single acoustic feature (32–53%). This could be due to the difference in the context, but other methodological differences could also contribute to the difference in the classification performance between the current and previous studies. In particular, the difference in the call sample size could contribute to the difference in the classification performance substantially. It would be interesting to compare our result with those obtained from animals in various contexts with a call sample size comparable to our study.

Neuronal mechanisms for integrating multiple acoustic cues for voice recognition may exist in the auditory cortex. There are auditory neurons sensitive to a combination of harmonically related tones [43–45]. In the macaque ventral auditory stream [46–48], the higher-order rostral auditory cortex is implicated in coding of caller identity from conspecific calls [49,50]. The rostral auditory cortex could achieve this by integrating spectral and temporal acoustic features to obtain a complex representation of conspecific vocalizations [25,51]. The integrated auditory voice information could then be combined with visual information of the caller in multisensory neurons found in the upper bank of the anterior superior temporal sulcus [50] through an identified functional coupling with the rostral auditory area [52].

Our results demonstrate a clear acoustic basis for caller identification from voice, robust across variable utterances. The result can guide investigation to determine the perceptual ability to integrate acoustic cues for speaker identification and the underlying neuronal mechanisms shared among humans and animals [53,54].
